# Mutation of the Glucosinolate Biosynthesis Enzyme Cytochrome P450 83A1 Monooxygenase Increases Camalexin Accumulation and Powdery Mildew Resistance

**DOI:** 10.3389/fpls.2016.00227

**Published:** 2016-03-02

**Authors:** Simu Liu, Lisa M. Bartnikas, Sigrid M. Volko, Frederick M. Ausubel, Dingzhong Tang

**Affiliations:** ^1^State Key Laboratory of Plant Cell and Chromosome Engineering, Institute of Genetics and Developmental Biology – Chinese Academy of SciencesBeijing, China; ^2^University of Chinese Academy of SciencesBeijing, China; ^3^Department of Molecular Biology, Massachusetts General Hospital, BostonMA, USA; ^4^Department of Genetics, Harvard Medical School, BostonMA, USA

**Keywords:** camalexin, powdery mildew, CYP83A1, plant immunity, *Arabidopsis thaliana*

## Abstract

Small secondary metabolites, including glucosinolates and the major phytoalexin camalexin, play important roles in immunity in *Arabidopsis thaliana*. We isolated an *Arabidopsis* mutant with increased resistance to the powdery mildew fungus *Golovinomyces cichoracearum* and identified a mutation in the gene encoding cytochrome P450 83A1 monooxygenase (CYP83A1), which functions in glucosinolate biosynthesis. The *cyp83a1-3* mutant exhibited enhanced defense responses to *G. cichoracearum* and double mutant analysis showed that this enhanced resistance requires NPR1, EDS1, and PAD4, but not SID2 or EDS5. In *cyp83a1-3* mutants, the expression of genes related to camalexin synthesis increased upon *G. cichoracearum* infection. Significantly, the *cyp83a1-3* mutant also accumulated higher levels of camalexin. Decreasing camalexin levels by mutation of the camalexin synthetase gene *PAD3* or the camalexin synthesis regulator *AtWRKY33* compromised the powdery mildew resistance in these mutants. Consistent with these observations, overexpression of *PAD3* increased camalexin levels and enhanced resistance to *G. cichoracearum.* Taken together, our data indicate that accumulation of higher levels of camalexin contributes to increased resistance to powdery mildew.

## Introduction

To protect themselves against pathogens, plants have evolved intricate immune responses that include accumulation of reactive oxygen species, deposition of callose, enhanced expression of *pathogenesis-related* (*PR*) genes, and biosynthesis of phytoalexins. Phytoalexins are low molecular mass secondary metabolites that are induced by both biotic and abiotic stress. During pathogen infection, plants synthesize a wide variety of structurally different phytoalexins to defend against pathogen invasion (Hammerschmidt, 1999; Pedras et al., 2011). Camalexin, 3-thiazol-2′-yl-indole, is one of the major phytoalexins of *Arabidopsis thaliana* and was long considered to be the only phytoalexin of *Arabidopsis*, until the discovery of rapalexin A (Pedras and Adio, 2008).

The biosynthesis and regulation of camalexin in *Arabidopsis* remain only partially understood, and the full scope of camalexin functions also remains to be defined. Camalexin is derived from tryptophan and requires many cytochrome P450s, including CYP79B2, CYP71A13, and CYP71B15 [which corresponds to the camalexin-deficient mutant *PHYTOALEXIN DEFICIENT 3* (*PAD3*); Nafisi et al., 2007; Schuhegger et al., 2007a,b]. The *Arabidopsis* transcription factors WRKY33, WRKY18, and WRKY40 appear to be involved in the regulation of camalexin biosynthesis (Qiu et al., 2008; Pandey et al., 2010; Mao et al., 2011). Camalexin plays an important role in the response to necrotrophic pathogens *Alternaria brassicicola* and *Botrytis cinerea* (Thomma et al., 1999; Ferrari et al., 2003; Kliebenstein et al., 2005; Nafisi et al., 2007), and the oomycete *Phytophthora brassicae* (Schlaeppi et al., 2010), as well as the biotrophic fungus *Golovinomyces orontii* (Consonni et al., 2010; Pandey et al., 2010). Although, camalexin produces broad-spectrum resistance to many species of plant pathogens, how it functions remains unclear.

In addition to camalexin, plants synthesize other, related secondary metabolites, such as glucosinolates, that participate in the defense response. Plant cells usually store glucosinolates in stable forms; during insect and/or pathogen attack, myrosinases hydrolyze these stable forms into active compounds (Halkier and Gershenzon, 2006). According to their side-chain radical, glucosinolates can be divided into aliphatic glucosinolates, indole glucosinolates, and aromatic glucosinolates. Many cytochrome P450s function in glucosinolate synthesis, including CYP83A1 and CYP83B1. The *Arabidopsis* cytochrome P450 monooxygenase CYP83A1 participates in the biosynthesis of aliphatic glucosinolates from aliphatic oximes, whereas CYP83B1, the *Arabidopsis* protein most similar to CYP83A1, functions in the biosynthesis of indole glucosinolates.

The biosynthetic pathways of alkylglucosinolates and indole glucosinolates affect each other; for instance, the *cyp83a1-2* (also called *ref2-1*) mutant produces lower levels of aliphatic glucosinolates, but accumulates higher levels of indole-derived glucosinolates compared with wild-type (Hemm et al., 2003; Naur et al., 2003; Sonderby et al., 2010). The biosynthesis of glucosinolates, especially indole glucosinolates, shares the intermediate product indole-3-acetaldoxime (IAOx) with biosynthetic pathways that produce many other secondary metabolites or hormones like camalexin and indole-3-acetic acid (IAA), respectively (Hemm et al., 2003; Grubb and Abel, 2006; Nafisi et al., 2007). Although the regulation of callose biosynthesis, in response to bacterial elicitors of *Arabidopsis* immunity, requires 4-methoxy-indol-3-ylmethylglucosinolate (4MI3G; Bednarek et al., 2009; Clay et al., 2009), how 4MI3G and related metabolites participate in the immune response is not well-understood.

Powdery mildew fungi, as biotrophic pathogens, infect many plant species and cause huge agricultural losses worldwide. The plant hormone salicylic acid (SA) plays an important role in resistance to powdery mildew in *Arabidopsis*, and many mutants showing enhanced resistance to powdery mildew require SA signaling for their resistance phenotype; these mutants include *edr1* (*enhanced disease resistance 1)*, *edr2* and *edr4* (Frye and Innes, 1998; Frye et al., 2001; Tang et al., 2005a,b; Zhao et al., 2014; Wu et al., 2015).

To further study resistance to powdery mildew in *Arabidopsis*, we characterized an *Arabidopsis* mutant that exhibits enhanced resistance to a variety of powdery mildew species. Here, we report that a mutation in the gene encoding cytochrome P450 monooxygenase CYP83A1, a component of the glucosinolate pathway, leads to higher accumulation of camalexin and enhanced resistance to the powdery mildew fungus *Golovinomyces cichoracearum*, which is consistent with the previous finding that *cyp83a1* exhibits increased resistance to powdery mildew fungus *Erysiphe cruciferarum* (Weis et al., 2013). We show that *cyp83a1-3* accumulates higher levels of camalexin. We also show that mutations in genes affecting camalexin production suppress the resistance of *cyp83a1-3*, indicating that higher accumulation of camalexin in *cyp83a1-3* mutants contributes to their enhanced powdery mildew resistance.

## Materials and Methods

### Isolation of the *cyp83a1-3* Mutant

The *cyp83a1-3* mutant was identified in a population of transgenic *Arabidopsis* Col-0 plants that expressed a *PR2::GUS* transgene and had been mutagenized with ethyl methanesulfonate as described (Cao et al., 1994). Two leaves of each of 3850 M2 plants were infiltrated with *Pseudomonas syringae* pv. *maculicola* strain ES4326 at a dose of 10^5^ cells per cm^2^ leaf area. Four putative mutants reproducibly exhibited reduced disease symptoms 3 days after infection, and two of these exhibited significantly reduced growth (about 10-fold less) of *P. syringae* ES4326. One of these latter two mutants, which was eventually named *cyp83a1-3*, also exhibited reduced symptom development when infected with the *G. orontii* strain MGH. The *cyp83a1-3* mutant was backcrossed twice to the parental line carrying the *PR2::GUS* reporter. Genetic analysis showed that the resistance phenotype of *cyp83a1-3* was recessive and segregated 1:3 as expected for a single recessive Mendelian gene.

### Plant Materials and Growth Conditions

*pad3-1* (Zhou et al., 1999), *cyp83a1-1* (Salk_123405), *cyp83a1-2* (*ref2-1*; Hemm et al., 2003) and *wrky33-2* (GABI_324B11; Zheng et al., 2006) were described previously. *Arabidopsis* plants were grown in a growth room at 20–22°C under a 9-h-light/15-h-dark cycle for phenotyping or a 16-h-light/8-h-dark cycle for seed setting, as described previously (Nie et al., 2011).

### Pathogen Infection and Microscopy

Powdery mildew pathogens *G. orontii* strain MGH (Plotnikova et al., 1998) and *G. cichoracearum* strain UCSC1 were maintained on *pad4-1* plants (Jirage et al., 1999) as described previously (Frye et al., 2001). Four-weeks-old plants were inoculated with powdery mildew using a settling tower to achieve an even distribution of conidia (Wang et al., 2011). To quantify fungal growth and conidiation, the number of conidiophores per colony was counted at 5 dpi (Consonni et al., 2006). At least 30 colonies were counted for each genotype in each experiment. Trypan blue staining was used to visualize fungal hyphae and dead cells (Frye and Innes, 1998), and H_2_O_2_ accumulation was detected with 3,3′-diaminobenzidine hydrochloride (DAB) staining (Koch and Slusarenko, 1990). Samples were photographed with an Olympus BX53 microscope.

### Map-Based Cloning and Complementation

For map-based cloning, we crossed the *cyp83a1-3* mutant with Landsberg *erecta* and the mutation was mapped using a variety of molecular markers to a 128-kb region on chromosome 4 spanned by two BAC clones, T6G15 and F18A5 (https://www.arabidopsis.org). This region contains 24 predicted genes. Using a candidate gene approach, genes in the region were amplified by PCR and sequenced until the mutation was identified as a single nucleotide change in *At4g13770*, which encodes the cytochrome P450 CYP83A1. The mutation results in the substitution of glutamic acid for a conserved glycine at amino acid position 346 in the heme-binding site of the enzyme.

The genomic DNA sequence of *CYP83A1* including 1.1 kb upstream of the ATG start codon and 0.4 kb downstream of the stop codon of *At4g13770* was cloned into binary vector pCambia1300 for complementation analysis. The derived genomic construct was verified by sequencing and introduced into *Agrobacterium tumefaciens* strain GV3101, then transformed into *cyp83a1-3* using the floral dip method (Clough and Bent, 1998). The transgenic plants were selected on 1/2 MS medium with 50 mg/L hygromycin.

### Construction of Double Mutants

The following mutants were crossed with *cyp83a1-3* to construct double mutants: *sid2-2* (Wildermuth et al., 2001), *pad4-1* (Jirage et al., 1999), *eds1-2* (Bartsch et al., 2006), *eds5-1* (Nawrath et al., 2002), *npr1-63* (Alonso et al., 2003), *pad3-1* (Zhou et al., 1999), and *wrky33-2* (Zheng et al., 2006). Double mutants were identified by PCR, except for the *pad3-1* mutation in *pad3-1 cyp83a1-3*, which was identified by PCR followed by sequencing.

### Quantitative Real-Time RT-PCR

Real-time quantitative PCR was performed as described previously (Nie et al., 2012).

### SA Extraction and Measurement

Salicylic acid extraction and measurement were performed as described previously (Gou et al., 2009).

### Vector Construction

The full-length *CYP83A1* coding sequence (CDS) without the stop codon was amplified by PCR from Col-0 cDNA and inserted into the Gateway vector pDONR207 using a BP Clonase kit (Invitrogen) to create a pDONR207-*CYP83A1* CDS entry clone. Then an LR Clonase kit (Invitrogen) was used to introduce the inserts into the pEarleyGate 101/103 destination vector containing a 35S promoter and C-terminal HA/GFP fusion (Earley et al., 2006). The same methods were used to construct *PAD3* overexpression constructs.

### Camalexin Measurement

Camalexin content was determined using a previously described fluorometric method (Glazebrook and Ausubel, 1994) with excitation at 315 nm and emission at 385 nm using a HITACHI F4500 spectrofluorometer. The concentration of camalexin was determined by comparison with a camalexin standard curve using purified camalexin kindly provided by Dr. Shuqun Zhang (University of Missouri).

### The Statistical Analysis

Statistical comparison of counts in genotypes in each of three independent experiments was performed using a mixed effects model for nested ANOVA, implemented in R. The genotypes were treated as fixed effects, whereas different experiments were treated as random effects. The resulting ANOVA P-values were used as estimates of statistical significance of the difference between genotypes.

### Gene ID Numbers

Sequence data from this article can be found in the *Arabidopsis* Genome Initiative databases under the following gene ID numbers: *Arabidopsis CYP83A1* (At4g13770), *PAD3* (At3g26 830), *CYP71A13* (At2g30770), *WRKY33* (At2g38470), *PR1* (At2g14610), *PR2* (At3g57260), *FRK1* (At2g19190), and *ACTIN2* (At3g18780).

Primers used for genotyping and gene expression analysis are listed in **Supplementary Table S1**.

## Results

### The *cyp83a1-3* Mutant Displays Reduced Susceptibility to the Powdery Mildew Fungi *G. orontii* and *G. cichoracearum*

To study the molecular mechanism of plant resistance to powdery mildew, we initially screened an ethyl methanesulfonate-mutagenized *Arabidopsis* ecotype Col-0 population for mutants with enhanced resistance to *P. syringae* pv. *maculicola* strain ES4326 and then subsequently for resistance to the powdery mildew fungi *G. orontii* and *G. cichoracearum*. In this screen (see Materials and Methods), we identified two mutants with enhanced resistance to *P. syringae*, one of which also showed increased resistance to powdery mildew. We designated this latter mutant *cyp83a1-3* based on subsequent characterization, as described below.

**FIGURE 1:**
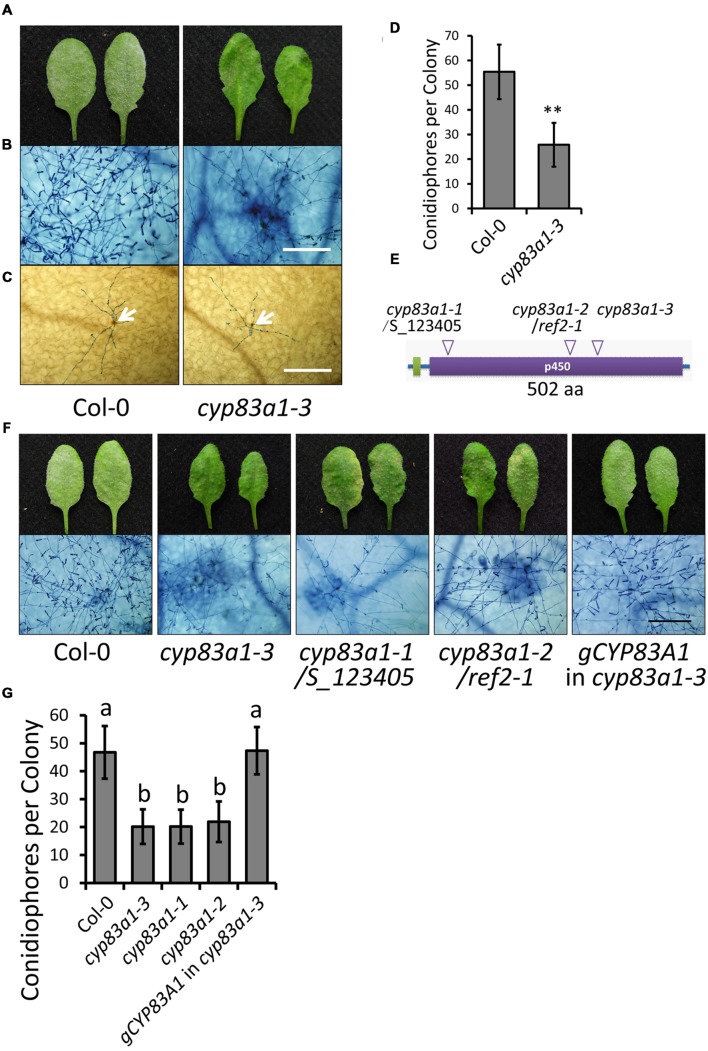
**The *cyp83a1-3* mutant displays enhanced resistance to *Golovinomyces cichoracearum*.** **(A)** Four-weeks-old *Arabidopsis* wild-type and *cyp83a1-3* mutant plants were infected with *G. cichoracearum*, and representative leaves were removed and photographed at 8 dpi. **(B)** Leaves were stained with trypan blue at 8 dpi, bar = 200 μm. **(C)** Leaves were stained with DAB and trypan blue at 2 dpi; arrows indicate H_2_O_2_ accumulation, bar = 200 μm. **(D)** Quantification of fungal growth in plants at 5 dpi by counting the number of conidiophores per colony. Results represent the mean and standard deviation in three independents experiments (*n* = 30). Asterisk represents statistically significant differences from wild-type (*P* < 0.01, nested ANOVA). **(E)** Schematic representation of the CYP83A1 protein, arrows indicate mutation sites of three *cyp83a1* mutant alleles. **(F)** Four-weeks-old wild-type, *cyp83a1-3*, *cyp83a1-1*, *cyp83a1-2*, and transgenic *cyp83a1-3* mutant plants complemented with the wild-type *CYP83A1* gene (*gCYP83A1*) were infected with *G. cichoracearum* and representative leaves were stained with trypan blue at 8 dpi, bar = 200 μm. **(G)** Quantification of fungal growth in plants at 5 dpi by counting the number of conidiophores per colony. Results represent the mean and standard deviation in three independent experiments (*n* = 30; *P* < 0.01, nested ANOVA).

In the absence of pathogen, the growth of the *cyp83a1-3* mutant was similar to the wild-type under standard short-day conditions (**Supplementary Figure S1**). However, *cyp83a1-3* mutants showed significantly fewer conidia with minor *G. cichoracearum*-induced lesions in comparison to wild-type plants at 8 days post-infection (dpi; **Figures 1A,B**). Powdery mildew infection often causes accumulation of H_2_O_2_ in resistant plants (Shi et al., 2013; Wu et al., 2015; Zhao et al., 2015). We used DAB staining to measure H_2_O_2_ accumulation at 2 dpi, but we found that the *cyp83a1-3* and wild-type plants had similar levels of H_2_O_2_ (**Figure 1C**), suggesting that an enhanced oxidative burst is not responsible for the resistance phenotype of the *cyp83a1-3* mutant. To quantitate the level of enhanced resistance of *cyp83a1-3* to *G. cichoracearum*, we quantified fungal growth by counting the number of conidiophores per colony and found that the *cyp83a1-3* mutant showed significantly fewer conidiophores per colony than the wild-type at 5 dpi (**Figure 1D**). These results are consistent with previous results showing that plants deficient in CYP83A1 are more resistant to a different powdery mildew species, *E. cruciferarum* (Weis et al., 2013).

To identify the mutation in *cyp83a1-3* responsible for the enhanced resistance phenotype, we carried out standard map-based cloning as described in Materials and Methods and identified a mutation (GA) in *At4g13770*, which encodes CYP83A1 and causes an amino acid substitution (G346E). The cytochrome P450 monooxygenase CYP83A1 functions in the biosynthesis of aliphatic glucosinolates from aliphatic oximes. Previous studies also identified mutations in the *CYP83A1* gene. The *cyp83a1-1* mutant (SALK_123405) contains a T-DNA insertion in the open reading frame of *CYP83A1* (Weis et al., 2013), and *cyp83a1-2*/*ref2-1* contains a loss-of-function point mutation leading to a premature stop codon (W58stop) in the *CYP83A1* gene (Hemm et al., 2003; **Figure 1E**).

To correlate the mutation in *cyp83a1-3* with the powdery mildew resistance phenotype, we cloned the wild-type *CYP83A1* gene driven by its native promoter and transformed this construct into the *cyp83a1-3* mutant. *Arabidopsis* wild-type Col-0 is susceptible to *G. cichoracearum.* This construct reversed the powdery mildew resistance phenotype in the *cyp83a1-3* mutant. In addition, the allelic mutants *cyp83a1-1* and *cyp83a1-2* showed similar *G. cichoracearum* resistant phenotypes as *cyp83a1-3* (**Figures 1F,G**). Taken together, these results indicate that the mutation in *CYP83A1* in *cyp83a1-3* causes the powdery mildew resistance phenotype.

### Resistance of *cyp83a1-3* to *G. cichoracearum* Does Not Require SA Signaling

**FIGURE 2:**
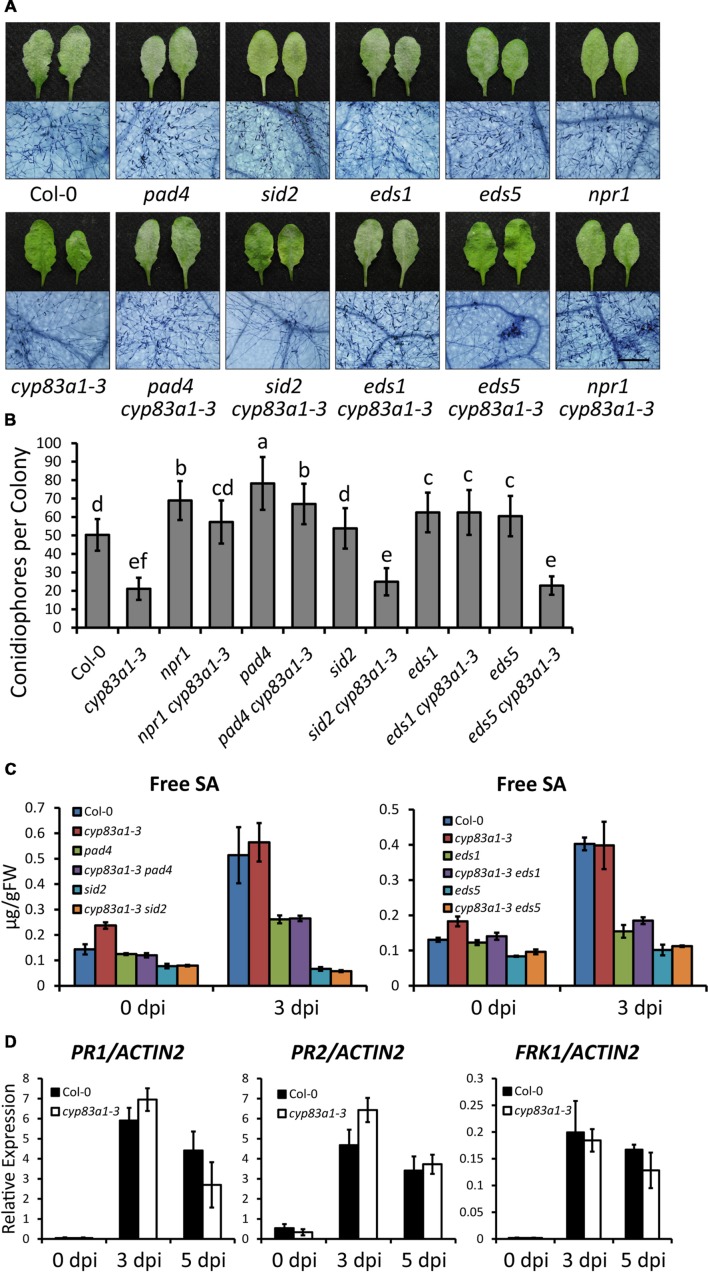
**The resistance in *cyp83a1-3* is SA-independent.** **(A)** Four-weeks-old *Arabidopsis* wild-type, *cyp83a1-3* mutant, and double mutant plants were infected with *G. cichoracearum*, and representative leaves were removed and stained with trypan blue at 8 dpi, bar = 200 μm. **(B)** Quantification of fungal growth of the plants in (a) at 5 dpi by counting the number of conidiophores per colony. Results represent the mean and standard deviation in three independent experiments (*n* = 30). Different letters represent statistically significant differences (*P* < 0.01, nested ANOVA). **(C)** Free SA levels were measured in the uninfected and infected (3 dpi) leaves after inoculation with *G. cichoracearum*. FW, fresh weight. **(D)** Accumulation of *PR1*, *PR2*, and *FRK1* transcripts in 4-weeks-old plants infected by *G. cichoracearum* examined by quantitative real-time PCR. Results represent the mean and standard deviation in three independent experiments (*n* = 4).

To investigate the cause of the resistance to *G. cichoracearum* in *cyp83a1-3*, we first examined whether the phytohormone SA, which plays an important role in resistance to biotrophic pathogens, is involved. We constructed double mutants by crossing *cyp83a1-3* with *sid2*, *eds5, npr1, pad4*, and *eds1*, well-characterized mutants with defects in SA accumulation or signaling. Double mutants were identified by PCR amplification (**Supplementary Figure S2**). We then inoculated the wild-type, single, and double mutants with *G. cichoracearum*, and performed trypan blue staining at 8 dpi. As shown in **Figure 2A**, the resistance of *cyp83a1-3* requires PAD4, EDS1, and NPR1, but not SID2 or EDS5. We also counted the number of conidiophores per colony in these plants, and the results were consistent with the staining assay (**Figure 2B**).

To further assess the role of SA in powdery mildew resistance in *cyp83a1-3*, we measured the accumulation of SA before and after *G. cichoracearum* infection. The *cyp83a1-3* mutant accumulated similar levels of SA as the wild-type at 3 dpi, and the levels of SA induced by powdery mildew were suppressed by mutations in *PAD4*, *SID2*, *EDS1, and EDS5* (**Figure 2C**). Mutation of *SID2* or *EDS5* suppressed SA accumulation in *cyp83a1-3*, but it did not suppress the resistance phenotype, indicating that the *cyp83a1-3* powdery mildew resistance phenotype may not require SA.

In addition, we measured the expression of defense-related genes, including *PR1* (*PATHOGENESIS-RELATED GENE1*), *PR2*, and *FRK1* (*FLG22-INDUCED RECEPTOR-LIKE KINASE1*), and found that the *cyp83a1-3* mutant accumulated similar levels of *PR1*, *PR2*, and *FRK1* transcripts as the wild-type at both 3 and 5 days after infection with *G. cichoracearum* (**Figure 2D**). Taken together, these results indicate that the resistance of *cyp83a1-3* to *G. cichoracearum* is dependent on PAD4, EDS1, and NPR1, but does not appear to be due to the increased accumulation of SA.

### The Expression of Genes Related to Camalexin Synthesis Increase upon *G. cichoracearum* Infection

To understand what causes powdery mildew resistance in *cyp83a1-3* mutants, we analyzed the secondary metabolic network that involves CYP83A1 (**Supplementary Figure S3**). Since the biosynthetic pathways for production of aliphatic glucosinolates and many indole-derived compounds are closely related, defects in CYP83A1 could change other indole-derived pathways and in turn affect powdery mildew resistance.

**FIGURE 3:**
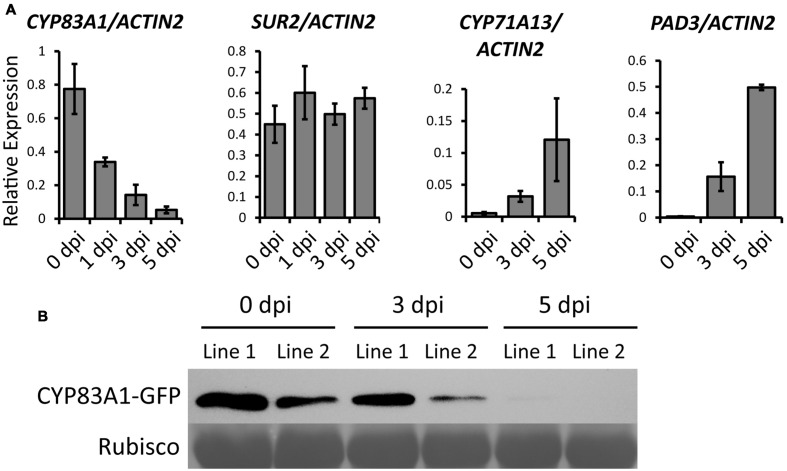
**Transcript accumulation of genes related to glucosinolate or camalexin biosynthesis upon *G. cichoracearum* infection.** **(A)** Four-weeks-old wild-type plants were infected with *G. cichoracearum*, and the accumulation of *CYP83A1*, *SUR2*, *CYP71A13*, and *PAD3* transcripts was examined by quantitative real-time PCR. Results represent the mean and standard deviation in three independent experiments (*n* = 4). **(B)** Four-weeks-old *CYP83A1-GFP* transgenic plants were infected with *G. cichoracearum* and immunoblot analysis was performed using an anti-GFP antibody. The large subunit of Rubisco was used as a protein loading control.

To investigate which alkylglucosinolate synthesis-related pathway contributes to the powdery mildew resistance, we first analyzed the expression of genes in three pathways, including the alkylglucosinolate pathway, indole glucosinolate pathway, and camalexin pathway, upon powdery mildew infection in Col-0 wild-type plants. We chose *CYP83A1* to represent the alkylglucosinolate pathway, *CYP83B1*/*SUR2* for the indole glucosinolate pathway, and *CYP71A13* and *CYP71B15*/*PAD3* for the camalexin pathway. We examined the expression of these genes using quantitative RT-PCR before and after *G. cichoracearum* infection. As shown in **Figure 3A**, the transcript levels of *CYP83A1* decreased, the transcript level of *SUR2* remained the same, but transcript levels of the camalexin synthetase genes *CYP71A13* and *PAD3* increased upon infection. These results suggested that camalexin may play an important role in the resistance to *G. cichoracearum* in *Arabidopsis*.

To examine whether CYP83A1 protein levels also decrease during powdery mildew infection, we constructed a plasmid to express a CYP83A1-GFP chimeric protein by fusing the genomic *CYP83A1* sequence to a C-terminal *GFP* sequence driven by the native *CYP83A1* promoter, and transformed it into the *cyp83a1-3* mutant. The transgene rescued the mutant phenotype (**Supplementary Figures S4A,B**), indicating that CYP83A1-GFP was functional. We inoculated the *CYP83A1-GFP* transgenic plants with powdery mildew and examined the accumulation of CYP83A1-GFP by immunoblot with a GFP antibody. As shown in **Figure 3B**, the CYP83A1 protein level decreased during infection, consistent with the observation that the *CYP83A1* mRNA level also decreased.

### The *cyp83a1-3* Mutant Accumulates High Levels of Camalexin and Resistance Requires the Camalexin Synthetase PAD3

**FIGURE 4:**
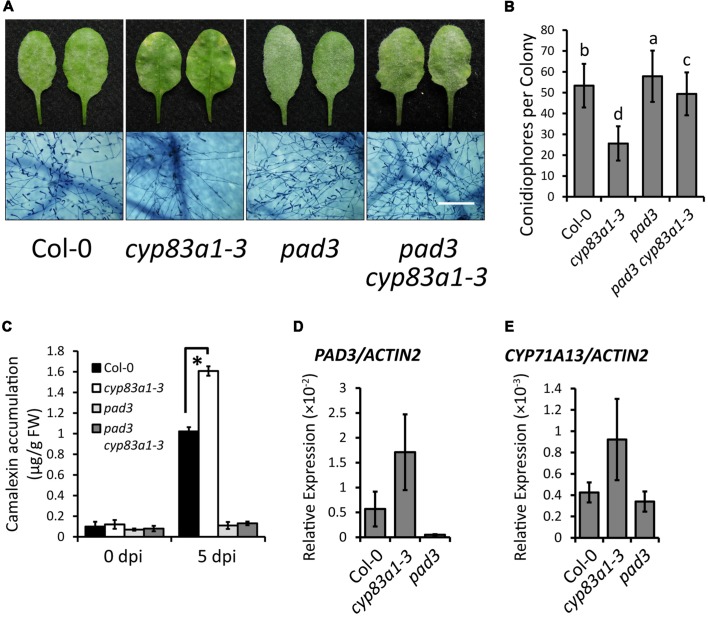
**The *cyp83a1-3* mutant accumulates high levels of camalexin upon *G. cichoracearum* infection, which is suppressed by mutation of *PAD3*.** **(A)** Four-weeks-old wild-type, *cyp83a1-3*, *pad3* mutant, and double-mutant plants were infected with *G. cichoracearum*. Representative leaves were removed and stained with trypan blue at 8 dpi, bar = 200 μm. **(B)** Quantification of fungal growth of the plants in (a) at 5 dpi by counting the number of conidiophores per colony. Results represent the mean and standard deviation in three independent experiments (*n* = 30; *P* < 0.01, nested ANOVA). **(C)** Four-weeks-old plants were infected with *G. cichoracearum*. Camalexin accumulation was determined at 0 and 5 dpi. Results represent the mean and standard deviation in three experiments (*n* = 3). Asterisk represents statistically significant difference from wild-type (*P* < 0.01, nested ANOVA). **(D)** The transcript accumulation of *PAD3* was examined by quantitative real-time PCR on samples from 4-weeks-old wild-type, *cyp83a1-3* and *pad3* mutant plants. **(E)** The transcript accumulation of *CYP71A13* examined by quantitative real-time PCR. Results represent the mean and standard deviation in three independent experiments (*n* = 4).

Since the biosynthetic pathways of glucosinolates and camalexin involve the same intermediates, and the expression of genes related to camalexin synthesis increase upon *G. cichoracearum* infection (**Figure 3A**), we hypothesized that increased levels of camalexin in the *cyp83a1-3* mutant compared to wild-type plants may be the reason that *cyp83a1-3* displays enhanced powdery mildew resistance. To test this hypothesis, we first constructed double mutants of *cyp83a1-3* with *pad3*, which accumulates lower levels of camalexin, compared with wild-type (Glazebrook and Ausubel, 1994). We then infected the double mutants with *G. cichoracearum*. As shown in **Figures 4A,B**, the *pad3* mutation suppressed the resistance phenotype of *cyp83a1-3*, indicating that the *cyp83a1-3* phenotype requires the PAD3 camalexin synthetase (CYP71B15).

To further assess the role of camalexin in *cyp83a1-3* resistance, we also measured the camalexin levels in *cyp83a1-3* before infection and at 5 dpi with *G. cichoracearum*. As shown in **Figure 4C**, after infection, the *cyp83a1-3* mutant accumulated significantly more camalexin than the wild-type, and this increase was suppressed by a mutation in *PAD3*. In addition, we found that the transcript levels of the camalexin synthesis genes *PAD3* and *CYP71A13* were higher in *cyp83a1-3* than in the wild-type in the absence of pathogen (**Figures 4D,E**).

Taken together, these results demonstrate that *cyp83a1-3* accumulates more camalexin compared with wild-type plants, and that the camalexin synthetase PAD3 is required for *cyp83a1-3*-mediated resistance, thus suggesting that the phytoalexin camalexin contributes to powdery mildew resistance.

### The Resistance in *cyp83a1-3* Mutants Requires the Camalexin Synthesis Regulator AtWRKY33

**FIGURE 5:**
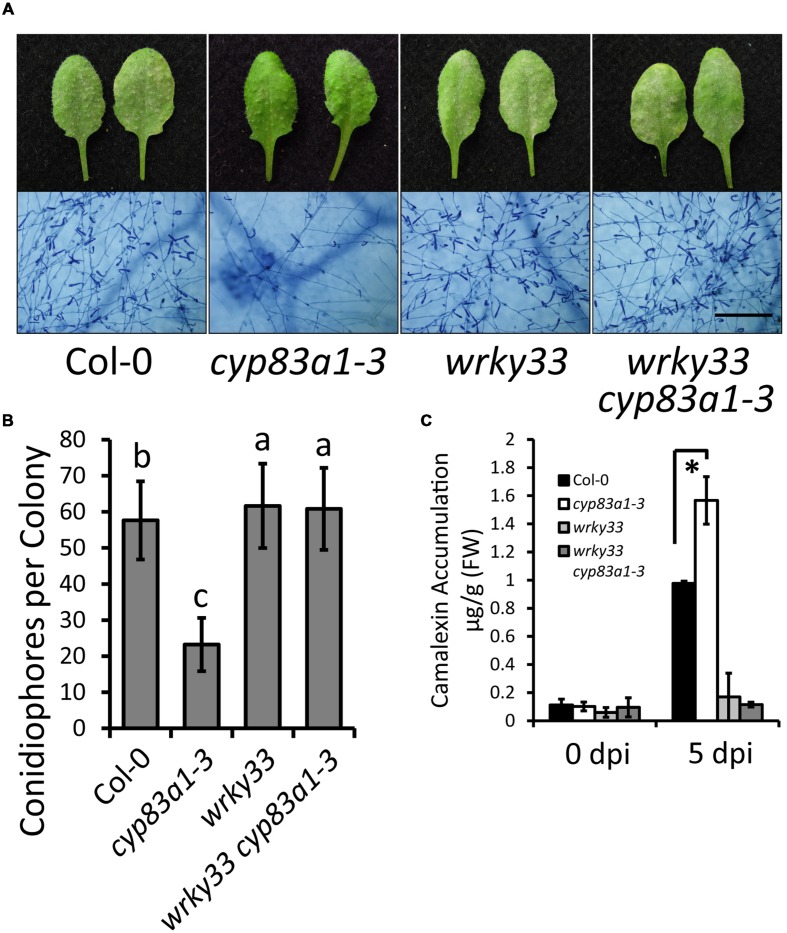
**The resistance phenotype and high levels of camalexin in *cyp83a1-3* is suppressed by mutation of *WRKY33*.** **(A)** Four-weeks-old wild-type, *cyp83a1-3*, *wrky33*, and *wrky33 cyp83a1-3* double-mutant plants were infected with *G. cichoracearum*. Representative leaves were removed and stained with trypan blue at 8 dpi, bar = 200 μm. **(B)** Quantification of fungal growth of the plants in (a) at 5 dpi by counting the number of conidiophores per colony. Results represent the mean and standard deviation in three independent experiments (*n* = 30; *P* < 0.01, nested ANOVA). **(C)** Camalexin accumulation of the plants in **(A)** was determined at 0 and 5 dpi. Results represent the mean and standard deviation in three independent experiments (*n* = 3). Asterisk represents statistically significant difference from wild-type (*P* < 0.01, nested ANOVA).

To further confirm the role of camalexin accumulation in *cyp83a1-3* resistance, we tested whether the resistance in *cyp83a1-3* mutants requires AtWRKY33, a transcription factor that positively regulates the expression of many camalexin synthetase genes. We constructed a *cyp83a1-3 wrky33* double mutant and examined the powdery mildew responses and camalexin levels in the single and double mutants. As shown in **Figures 5A–C**, the *wrky33* mutation not only suppressed powdery mildew resistance, but it also decreased camalexin accumulation in the *cyp83a1-3* mutant.

### Overexpression of *PAD3* Leads to Increased Camalexin Accumulation and Enhanced Resistance to *G. cichoracearum*

**FIGURE 6:**
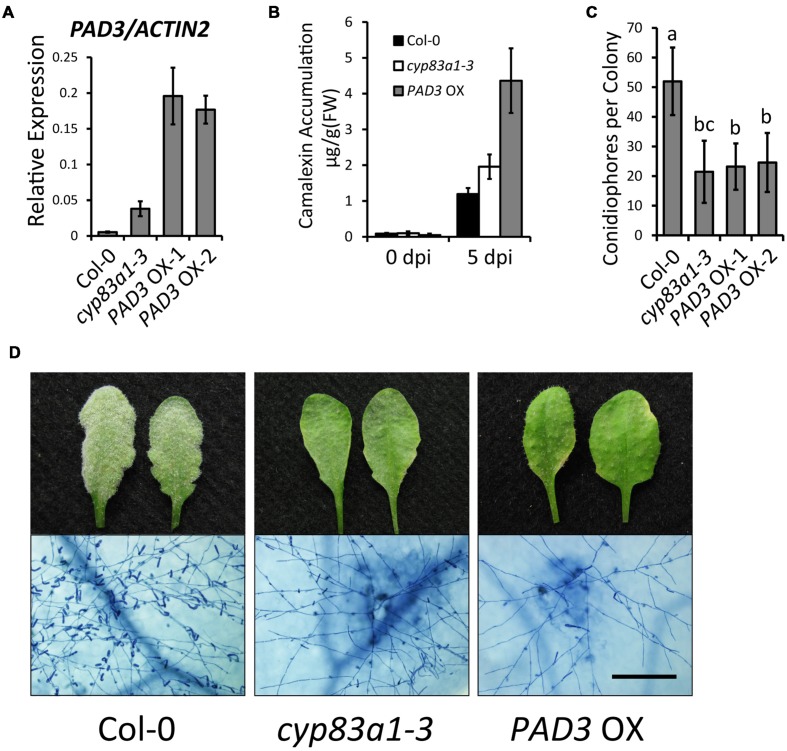
***PAD3*-overexpressing plants accumulate higher levels of camalexin and display a *cyp83a1-3*-like resistance phenotype.** **(A)** The accumulation of *PAD3* transcript in 4-weeks-old plants was examined by quantitative real-time PCR. Results represent the mean and standard deviation in three independent experiments (*n* = 4). **(B)** Four-weeks-old plants were infected with *G. cichoracearum* and camalexin accumulation was determined at 0 and 5 dpi. Three *PAD3-*OX independent lines were tested, and similar results were obtained in these lines. One representative *PAD3-*OX line (*PAD3-*OX*-1*) is shown. Results represent the mean and standard deviation in three experiments (*n* = 3). **(C)** Quantification of fungal growth of wild-type, *cyp83a1-3*, and *PAD3*-OX plants at 5 dpi by counting the number of conidiophores per colony. Results represent the mean and standard deviation in three independent experiments (*n* = 30). Different letters represent statistically significant differences (*P* < 0.01, nested ANOVA). **(D)** Four-weeks-old wild-type, *cyp83a1-3*, and *PAD3*-OX-1 plants were infected with *G. cichoracearum*. Three *PAD3-*OX independent lines were examined, and similar results were obtained in these three lines. Representative leaves were removed and stained with trypan blue at 8 dpi, bar = 200 μm.

To further confirm the role of camalexin in powdery mildew resistance, we constructed transgenic plants that overexpress *PAD3* (*PAD3*-OX). For this, we made a construct with the *PAD3* coding sequence driven by the 35S promoter and transformed this construct into wild-type plants. We used quantitative RT-PCR to measure the *PAD3* expression levels of two independent lines of T3 generation *PAD3*-OX plants (**Figure 6A**). After *G. cichoracearum* infection, the *PAD3-*OX plants showed higher camalexin accumulation at 5 dpi, compared with wild-type (**Figure 6B**). The *PAD3*-OX plants also displayed enhanced resistance to powdery mildew compared with the wild-type (**Figures 6C,D**), similar to the *cyp83a1-3* mutant. These results provide further evidence that higher levels of camalexin cause enhanced resistance to *G. cichoracearum*. Taken together, our findings revealed that the resistance to *G. cichoracearum* in *cyp83a1-3* is at least partially due to higher levels of camalexin accumulation.

## Discussion

The *cyp83a1-3* mutant displays enhanced resistance to powdery mildews, including *G. cichoracearum*. The levels of SA accumulation and *PR* gene expression in *cyp83a1-3* mutants were similar to the wild-type, and double mutant analysis showed that resistance in *cyp83a1-3* was dependent on *PAD4*, *EDS1*, and *NPR1* but independent of *SID2* and *EDS5*. Mutations in *SID2* or *EDS5* suppressed SA accumulation induced by powdery mildew but did not suppress the disease resistance, indicating that resistance in *cyp83a1-3* is not caused by enhanced SA signaling. These observations indicate that *cyp83a1-3*-mediated resistance differs from that in the *edr1* and *edr2* mutants (Frye et al., 2001; Tang et al., 2005b). Moreover, the *cyp83a1-3* mutant accumulates more camalexin upon powdery mildew infection, and mutations in *PAD3* or *WRKY33* suppressed both the disease resistance and the high accumulation of camalexin, indicating a link between camalexin levels and responses to powdery mildew. Consistent with a role of camalexin in powdery mildew resistance, the *PAD3*-overexpressing plants accumulated more camalexin and mimicked the resistance phenotype of the *cyp83a1-3* mutant. Taken together, those findings indicate that the higher level of camalexin contributes to the enhanced resistance to *G. cichoracearum* observed in the *cyp83a1-3* mutant. Our finding that *eds1* and *pad4* mutations suppressed powdery mildew resistance in *cyp83a1* is consistent with previous work showing that *eds1* and *pad4* have reduced levels of camalexin (Glazebrook et al., 1997; Mert-Türk et al., 2003). It would be interesting to examine whether NPR1 contributes to camalexin accumulation since NPR1 is also required for powdery mildew resistance in *cyp83a1*.

Our finding that camalexin plays an important role in powdery mildew resistance is also consistent with previous work showing that loss-of-function mutations in two transcription factors, WRKY18 and WRKY40, results in the accumulation of higher levels of camalexin as well as both preinvasive and post-invasive resistance against the powdery mildew fungus *G. orontii* (Pandey et al., 2010; Schön et al., 2013). Similar to our results, these authors also found that PAD3 (a key enzyme in camalexin biosynthesis) is required for the preinvasive resistance (but surprisingly not for the post-invasive resistance) against *G. orontii* in a *wrky18 wrky40* background (Schön et al., 2013). Moreover, Schön et al. (2013) also report that *wrky18 wrky40* plants do not show increased resistance against two other powdery mildews, *G. cichoracearum* and *G. cruciferarum.* The reason why we observed enhanced resistance to *G. cichoracearum* in *cyp83a1-3* whereas Schön et al. (2013) did not observe enhanced resistance in *wrky18 wrky40* is not clear, but may be related to differences in camalexin levels in the two mutants or to differences in susceptibility of different *G. cichoracearum* isolates to camalexin.

The function of CYP83A1 has been studied previously. An earlier study showed that overexpression of *CYP83A1* could rescue the auxin-excess phenotype of *cyp83b1*/*rnt1*/*sur2* mutants (Bak and Feyereisen, 2001). Further studies showed that levels of many phenylpropanoid pathway-derived products were reduced in the *cyp83a1-2*/*ref2-1* mutant, indicating crosstalk between the pathways producing aliphatic glucosinolates and indole glucosinolates (Hemm et al., 2003; Naur et al., 2003). Recent work showed that CYP83A1 interacts with BAX INHIBITOR-1, a cell death suppressor in plants and animals. The loss-of-function mutants *cyp83a1-1* and *cyp83a1-2* displayed enhanced resistance to the powdery mildew fungus *E. cruciferarum* (Weis et al., 2013). A more recent study measured the levels of several glucosinolates in *cyp83a1-1* mutants, but found only marginally increased amounts of indole-derived glucosinolates. The *cyp83a1* mutants lack very-long-chain aldehydes and accumulate more 5-methylthiopentanaldoxime (5-MPTO), a potentially toxic substrate of CYP83A1 (Weis et al., 2014). As very-long-chain aldehydes promote germination and appressorium formation of *E. cruciferarum*, it was proposed that lack of very-long-chain aldehydes causes the resistance phenotypes in *cyp83a1* mutants (Weis et al., 2014).

Here, we showed that the high level of camalexin contributes to resistance to the powdery mildew fungus *G. cichoracearum*. It is worth noting that different species of powdery mildew, *G. cichoracearum* and *E. cruciferarum*, were used in our study and in the Weis et al. (2014) study, respectively. Although *cyp83a1* mutants displayed enhanced resistance to both powdery mildew strains, the mechanisms could differ. Consistent with this notion, several studies observed differences in infection phenotypes between different powdery mildew species in *Arabidopsis*. For example, *G. cichoracearum* and *G. orontii* have different host ranges/responses (Plotnikova et al., 1998) and many *Arabidopsis* accessions show different responses to the powdery mildew species *E. cruciferarum* UEA1 and *G. cichoracearum* UCSC1 (Adam et al., 1999). Here, we showed that *pad3* and *wrky33* suppressed the accumulation of camalexin and the enhanced resistance in *cyp83a1-3* mutants, indicating a role of camalexin in *cyp83a1-3*-mediated resistance. It would be interesting to examine the responses of *pad3 cyp83a1-3* and *wrky33 cyp83a1-3* mutants to *E. cruciferarum*, and to measure the levels of very-long-chain aldehydes and 5-MPTO in those mutants. It is also possible that both very-long-chain aldehydes and camalexin contribute to resistance against to *G. cichoracearum* and *E. cruciferarum*.

CYP83A1 functions in the biosynthesis of aliphatic glucosinolates, so one interesting question is how the mutation of the glucosinolate synthetase gene *CYP83A1* affects the accumulation of camalexin. One explanation is that it may cause crosstalk within the complicated metabolic network. In this scenario, the biosynthetic pathway of aliphatic glucosinolates, which involves CYP83A1, and indole glucosinolates, which share the IAOx intermediate with camalexin (Nafisi et al., 2007; Schuhegger et al., 2007a; Bottcher et al., 2009), can affect each other. So when the aliphatic glucosinolates pathway is blocked in the *cyp83a1* mutant, the pathway for indole-derived products, including indole glucosinolates and camalexin, is enhanced. Indole glucosinolates are known to contribute to plant immunity (Bednarek, 2012). *Arabidopsis* PENETRATION2 (PEN2), which initiates indole glucosinolate metabolism, plays an important role in penetration resistance (Bednarek et al., 2009; Clay et al., 2009). In addition to increased levels of camalexin, the *cyp83a1* mutant has several other aberrant phenotypes including decreased levels of very-long-chain aldehydes and alterations in many metabolites that could also contribute to resistance, so the exact mechanism of powdery mildew resistance in *cyp83a1* needs to be further studied. Consistent with this notion, *PAD3*-overexpression plants accumulate much higher levels of camalexin than the *cyp83a1-3* mutant, but no further increase in powdery mildew resistance was observed. It would be interesting to measure very-long-chain aldehydes and indole-derived products in *pad3 cyp83a1* double mutants to further examine whether altered camalexin levels or differences in other metabolites are responsible for the powdery mildew resistance phenotype in *cyp83a1*.

## Conclusion

We showed that camalexin plays an important role in resistance to the powdery mildew pathogen *G. cichoracearum*. Our study provides new insights into the role of camalexin in plant immunity against powdery mildew.

## Author Contributions

DT, SL, and FA designed the project. SL, LB, and SV performed experiments. SL, LB, SV, FA, and DT discussed and interpreted results. SL and DT wrote the manuscript. All authors carefully read and edited the manuscript.

## Conflict of Interest Statement

The authors declare that the research was conducted in the absence of any commercial or financial relationships that could be construed as a potential conflict of interest.

## Supplementary Material

**FIGURE S1 | The growth phenotype of the *cyp83a1-3* mutant is similar to wild-type in the absence of *G. cichoracearum*.** Wild-type and *cyp83a1-3* mutants were grown under standard short day conditions. Five-weeks-old plants were photographed.

**FIGURE S2 | Identification of double mutants by PCR amplification.** The *pad4-1 cyp83a1-3*, *sid2-2 cyp83a1-3*, *eds1-2 cyp83a1-3 eds5-1 cyp83a1-3*, *npr1-63 cyp83a1-3*, and *wrky33-2 cyp83a1-3* mutants were identified by PCR amplification.

**FIGURE S3 | The biosynthetic network of glucosinolates and related compounds in *Arabidopsis*.** Schematic representation of the biosynthesis pathway derived from tryptophan and methionine.

**FIGURE S4 | *CYP83A1-GFP* rescued the powdery mildew-resistant phenotype of *cyp83a1-3*.** **(A)** Four-weeks-old wild-type, *cyp83a1-3*, and *cyp83a1-3* transgenic plants expressing the CYP83A1-GFP fusion protein driven by native promoter were infected with *G. cichoracearum*. Representative leaves were removed (upper panel) and stained with trypan blue (lower panel) at 8 dpi, bar = 200 μm. **(B)** Quantification of fungal growth of the plants in (a) at 5 dpi by counting the number of conidiophores per colony. Results represent the mean and standard deviation of three independent experiments (*n* = 30). Different letters represent statistically significant differences (*P* < 0.01, nested ANOVA).

## References

[B1] Adam L. Ellwood S. Wilson I. Saenz G. Xiao S. Oliver R. P. (1999). Comparison of *Erysiphe cichoracearum* and *E. cruciferarum* and a survey of 360 *Arabidopsis thaliana* accessions for resistance to these two powdery mildew pathogens. *Mol. Plant-Microbe Interact.* 12 1031–1043. 10.1094/MPMI.1999.12.12.1031 10624012

[B2] Alonso J. M. Stepanova A. N. Leisse T. J. Kim C. J. Chen H. Shinn P. (2003). Genome-wide insertional mutagenesis of *Arabidopsis thaliana*. *Science* 301 653–657. 10.1126/science.1086391 12893945

[B3] Bak S. Feyereisen R. (2001). The involvement of two p450 enzymes, CYP83B1 and CYP83A1, in auxin homeostasis and glucosinolate biosynthesis. *Plant Physiol.* 127 108–118. 10.1104/pp.127.1.108 11553739 PMC117967

[B4] Bartsch M. Gobbato E. Bednarek P. Debey S. Schultze J. L. Bautor J. (2006). Salicylic acid-independent ENHANCED DISEASE SUSCEPTIBILITY1 signaling in *Arabidopsis* immunity and cell death is regulated by the monooxygenase FMO1 and the nudix hydrolase NUDT7. *Plant Cell* 18 1038–1051. 10.1105/tpc.105.039982 16531493 PMC1425861

[B5] Bednarek P. (2012). Chemical warfare or modulators of defence responses - the function of secondary metabolites in plant immunity. *Curr. Opin. Plant Biol.* 15 407–414. 10.1016/j.pbi.2012.03.002 22445190

[B6] Bednarek P. Pislewska-Bednarek M. Svatos A. Schneider B. Doubsky J. Mansurova M. (2009). A glucosinolate metabolism pathway in living plant cells mediates broad-spectrum antifungal defense. *Science* 323 101–106. 10.1126/science.1163732 19095900

[B7] Bottcher C. Westphal L. Schmotz C. Prade E. Scheel D. Glawischnig E. (2009). The multifunctional enzyme CYP71B15 (PHYTOALEXIN DEFICIENT3) converts cysteine-indole-3-acetonitrile to camalexin in the indole-3-acetonitrile metabolic network of *Arabidopsis thaliana*. *Plant Cell* 21 1830–1845. 10.1105/tpc.109.066670 19567706 PMC2714930

[B8] Cao H. Bowling S. A. Gordon A. S. Dong X. N. (1994). Characterization of an *Arabidopsis* mutant that is nonresponsive to inducers of systemic acquired-resistance. *Plant Cell* 6 1583–1592. 10.1105/tpc.6.11.1583 12244227 PMC160545

[B9] Clay N. K. Adio A. M. Denoux C. Jander G. Ausubel F. M. (2009). Glucosinolate metabolites required for an *Arabidopsis* innate immune response. *Science* 323 95–101. 10.1126/science.1164627 19095898 PMC2630859

[B10] Clough S. J. Bent A. F. (1998). Floral dip: a simplified method for *Agrobacterium*-mediated transformation of *Arabidopsis thaliana*. *Plant J.* 16 735–743. 10.1046/j.1365-313x.1998.00343.x 10069079

[B11] Consonni C. Bednarek P. Humphry M. Francocci F. Ferrari S. Harzen A. (2010). Tryptophan-derived metabolites are required for antifungal defense in the *Arabidopsis* mlo2 mutant. *Plant Physiol.* 152 1544–1561. 10.1104/pp.109.147660 20023151 PMC2832281

[B12] Consonni C. Humphry M. E. Hartmann H. A. Livaja M. Durner J. Westphal L. (2006). Conserved requirement for a plant host cell protein in powdery mildew pathogenesis. *Nat. Genet.* 38 716–720. 10.1038/ng1806 16732289

[B13] Earley K. W. Haag J. R. Pontes O. Opper K. Juehne T. Song K. M. (2006). Gateway-compatible vectors for plant functional genomics and proteomics. *Plant J.* 45 616–629. 10.1111/j.1365-313X.2005.02617.x 16441352

[B14] Ferrari S. Plotnikova J. M. De Lorenzo G. Ausubel F. M. (2003). *Arabidopsis* local resistance to *Botrytis cinerea* involves salicylic acid and camalexin and requires EDS4 and PAD2, but not SID2, EDS5 or PAD4. *Plant J.* 35 193–205. 10.1046/j.1365-313X.2003.01794.x 12848825

[B15] Frye C. A. Innes R. W. (1998). An *Arabidopsis* mutant with enhanced resistance to powdery mildew. *Plant Cell* 10 947–956. 10.1105/tpc.10.6.947 9634583 PMC144036

[B16] Frye C. A. Tang D. Innes R. W. (2001). Negative regulation of defense responses in plants by a conserved MAPKK kinase. *Proc. Natl. Acad. Sci. U.S.A.* 98 373–378. 10.1073/pnas.011405198 11114160 PMC14597

[B17] Glazebrook J. Ausubel F. M. (1994). Isolation of phytoalexin-deficient mutants of *Arabidopsis thaliana* and characterization of their interactions with bacterial pathogens. *Proc. Natl. Acad. Sci. U.S.A.* 91 8955–8959. 10.1073/pnas.91.19.8955 8090752 PMC44725

[B18] Glazebrook J. Zook M. Mert F. Kagan I. Rogers E. E. Crute I. R. (1997). Phytoalexin-deficient mutants of *Arabidopsis* reveal that PAD4 encodes a regulatory factor and that four PAD genes contribute to downy mildew resistance. *Genetics* 146 381–392. 9136026 10.1093/genetics/146.1.381 PMC1207952

[B19] Gou M. Y. Su N. Zheng J. Huai J. L. Wu G. H. Zhao J. F. (2009). An F-box gene, CPR30, functions as a negative regulator of the defense response in *Arabidopsis*. *Plant J.* 60 757–770. 10.1111/j.1365-313X.2009.03995.x 19682297

[B20] Grubb C. D. Abel S. (2006). Glucosinolate metabolism and its control. *Trends Plant Sci.* 11 89–100. 10.1016/j.tplants.2005.12.006 16406306

[B21] Halkier B. A. Gershenzon J. (2006). Biology and biochemistry of glucosinolates. *Annu. Rev. Plant Biol.* 57 303–333. 10.1146/annurev.arplant.57.032905.105228 16669764

[B22] Hammerschmidt R. (1999). Phytoalexins: what have we learned after 60 years? *Annu. Rev. Phytopathol.* 37 285–306. 10.1146/annurev.phyto.37.1.285 11701825

[B23] Hemm M. R. Ruegger M. O. Chapple C. (2003). The *Arabidopsis* ref2 mutant is defective in the gene encoding CYP83A1 and shows both phenylpropanoid and glucosinolate phenotypes. *Plant Cell* 15 179–194. 10.1105/tpc.006544 12509530 PMC143490

[B24] Jirage D. Tootle T. L. Reuber T. L. Frost L. N. Feys B. J. Parker J. E. (1999). *Arabidopsis thaliana* PAD4 encodes a lipase-like gene that is important for salicylic acid signaling. *Proc. Natl. Acad. Sci. U.S.A.* 96 13583–13588. 10.1073/pnas.96.23.13583 10557364 PMC23991

[B25] Kliebenstein D. J. Rowe H. C. Denby K. J. (2005). Secondary metabolites influence *Arabidopsis*/*Botrytis* interactions: variation in host production and pathogen sensitivity. *Plant J.* 44 25–36. 10.1111/j.1365-313X.2005.02508.x 16167893

[B26] Koch E. Slusarenko A. (1990). *Arabidopsis* is susceptible to infection by a downy mildew fungus. *Plant Cell* 2 437–445. 10.1105/tpc.2.5.437 2152169 PMC159900

[B27] Mao G. Meng X. Liu Y. Zheng Z. Chen Z. Zhang S. (2011). Phosphorylation of a WRKY transcription factor by two pathogen-responsive MAPKs drives phytoalexin biosynthesis in *Arabidopsis*. *Plant Cell* 23 1639–1653. 10.1105/tpc.111.084996 21498677 PMC3101563

[B28] Mert-Türk F. Bennett M. H. Mansfield J. W. Holub E. B. (2003). Camalexin accumulation in *Arabidopsis thaliana* following abiotic elicitation or inoculation with virulent or avirulent *Hyaloperonospora parasitica*. *Physiol. Mol. Plant Pathol.* 62 137–145. 10.1016/s0885-5765(03)00047-x

[B29] Nafisi M. Goregaoker S. Botanga C. J. Glawischnig E. Olsen C. E. Halkier B. A. (2007). *Arabidopsis* cytochrome P450 monooxygenase 71A13 catalyzes the conversion of indole-3-acetaldoxime in camalexin synthesis. *Plant Cell* 19 2039–2052. 10.1105/tpc.107.051383 17573535 PMC1955726

[B30] Naur P. Petersen B. L. Mikkelsen M. D. Bak S. Rasmussen H. Olsen C. E. (2003). CYP83A1 and CYP83B1, two nonredundant cytochrome P450 enzymes metabolizing oximes in the biosynthesis of glucosinolates in *Arabidopsis*. *Plant Physiol.* 133 63–72. 10.1104/pp.102.019240 12970475 PMC196579

[B31] Nawrath C. Heck S. Parinthawong N. Metraux J. P. (2002). EDS5, an essential component of salicylic acid-dependent signaling for disease resistance in *Arabidopsis*, is a member of the MATE transporter family. *Plant Cell* 14 275–286. 10.1105/tpc.010376 11826312 PMC150564

[B32] Nie H. Wu Y. Yao C. Tang D. (2011). Suppression of edr2-mediated powdery mildew resistance, cell death and ethylene-induced senescence by mutations in ALD1 in *Arabidopsis*. *J. Genet. Genomics* 38 137–148. 10.1016/j.jgg.2011.03.001 21530897

[B33] Nie H. Zhao C. Wu G. Wu Y. Chen Y. Tang D. (2012). SR1, a calmodulin-binding transcription factor, modulates plant defense and ethylene-induced senescence by directly regulating NDR1 and EIN3. *Plant Physiol.* 158 1847–1859. 10.1104/pp.111.192310 22345509 PMC3320190

[B34] Pandey S. P. Roccaro M. Schon M. Logemann E. Somssich I. E. (2010). Transcriptional reprogramming regulated by WRKY18 and WRKY40 facilitates powdery mildew infection of *Arabidopsis*. *Plant J.* 64 912–923. 10.1111/j.1365-313X.2010.04387.x 21143673

[B35] Pedras M. S. C. Adio A. M. (2008). Phytoalexins and phytoanticipins from the wild crucifers *Thellungiella halophila* and *Arabidopsis thaliana*: rapalexin A, wasalexins and camalexin. *Phytochemistry* 69 889–893. 10.1016/j.phytochem.2007.10.032 18078965

[B36] Pedras M. S. C. Yaya E. E. Glawischnig E. (2011). The phytoalexins from cultivated and wild crucifers: chemistry and biology. *Nat. Prod. Rep.* 28 1381–1405. 10.1039/C1np00020a 21681321

[B37] Plotnikova J. M. Reuber T. L. Ausubel F. M. (1998). Powdery mildew pathogenesis of *Arabidopsis thaliana*. *Mycologia* 90 1009–1016. 10.2307/3761274

[B38] Qiu J. L. Fiil B. K. Petersen K. Nielsen H. B. Botanga C. J. Thorgrimsen S. (2008). *Arabidopsis* MAP kinase 4 regulates gene expression through transcription factor release in the nucleus. *EMBO J.* 27 2214–2221. 10.1038/emboj.2008.147 18650934 PMC2519101

[B39] Schlaeppi K. Abou-Mansour E. Buchala A. Mauch F. (2010). Disease resistance of *Arabidopsis* to *Phytophthora brassicae* is established by the sequential action of indole glucosinolates and camalexin. *Plant J.* 62 840–851. 10.1111/j.1365-313X.2010.04197.x 20230487

[B40] Schön M. Töller A. Diezel C. Roth C. Westphal L. Wiermer M. (2013). Analyses of wrky18 wrky40 plants reveal critical roles of SA/EDS1 signaling and indole-glucosinolate biosynthesis for *Golovinomyces orontii* resistance and a loss-of resistance towards *Pseudomonas syringae* pv. tomato AvrRPS4. *Mol. Plant-Microbe Interact.* 26 758–767. 10.1094/MPMI-11-12-0265-R 23617415

[B41] Schuhegger R. Nafisi M. Mansourova M. Petersen B. L. Olsen C. E. Svatos A. (2007a). CYP71B15 (PAD3) catalyzes the final step in camalexin biosynthesis - Correction. *Plant Physiol.* 145 1086–1086. 10.1104/pp.104.900240 PMC1533948 16766671

[B42] Schuhegger R. Rauhut T. Glawischnig E. (2007b). Regulatory variability of camalexin biosynthesis. *J. Plant Physiol.* 164 636–644. 10.1016/j.jplph.2006.04.012 16769150

[B43] Shi H. Shen Q. Qi Y. Yan H. Nie H. Chen Y. (2013). BR-SIGNALING KINASE_1_ physically associates with FLAGELLIN SENSING_2_ and regulates plant innate immunity in *Arabidopsis*. *Plant Cell* 25 1143–1157. 10.1105/tpc.112.107904 23532072 PMC3634682

[B44] Sonderby I. E. Geu-Flores F. Halkier B. A. (2010). Biosynthesis of glucosinolates–gene discovery and beyond. *Trends Plant Sci.* 15 283–290. 10.1016/j.tplants.2010.02.005 20303821

[B45] Tang D. Ade J. Frye C. A. Innes R. W. (2005a). Regulation of plant defense responses in *Arabidopsis* by EDR2, a PH and START domain-containing protein. *Plant J.* 44 245–257. 10.1111/j.1365-313X.2005.02523.x 16212604 PMC1797612

[B46] Tang D. Christiansen K. M. Innes R. W. (2005b). Regulation of plant disease resistance, stress responses, cell death, and ethylene signaling in *Arabidopsis* by the EDR1 protein kinase. *Plant Physiol.* 138 1018–1026. 10.1104/pp.105.060400 15894742 PMC1150416

[B47] Thomma B. P. H. J. Nelissen I. Eggermont K. Broekaert W. F. (1999). Deficiency in phytoalexin production causes enhanced susceptibility of *Arabidopsis thaliana* to the fungus *Alternaria brassicicola*. *Plant J.* 19 163–171. 10.1046/j.1365-313X.1999.00513.x 10476063

[B48] Wang Y. Nishimura M. T. Zhao T. Tang D. (2011). ATG2, an autophagy-related protein, negatively affects powdery mildew resistance and mildew-induced cell death in *Arabidopsis*. *Plant J.* 68 74–87. 10.1111/j.1365-313X.2011.04669.x 21645148

[B49] Weis C. Hildebrandt U. Hoffmann T. Hemetsberger C. Pfeilmeier S. Konig C. (2014). CYP83A1 is required for metabolic compatibility of *Arabidopsis* with the adapted powdery mildew fungus *Erysiphe cruciferarum*. *New Phytol.* 202 1310–1319. 10.1111/nph.12759 24602105

[B50] Weis C. Pfeilmeier S. Glawischnig E. Isono E. Pachl F. Hahne H. (2013). Co-immunoprecipitation-based identification of putative BAX INHIBITOR-1-interacting proteins involved in cell death regulation and plant-powdery mildew interactions. *Mol. Plant Pathol.* 14 791–802. 10.1111/mpp.12050 23782494 PMC6638788

[B51] Wildermuth M. C. Dewdney J. Wu G. Ausubel F. M. (2001). Isochorismate synthase is required to synthesize salicylic acid for plant defence. *Nature* 414 562–565. 10.1038/35107108 11734859

[B52] Wu G. Liu S. Zhao Y. Wang W. Kong Z. Tang D. (2015). ENHANCED DISEASE RESISTANCE4 associates with CLATHRIN HEAVY CHAIN2 and modulates plant immunity by regulating relocation of EDR1 in *Arabidopsis*. *Plant Cell* 27 857–873. 10.1105/tpc.114.134668 25747881 PMC4558660

[B53] Zhao C. Nie H. Shen Q. Zhang S. Lukowitz W. Tang D. (2014). EDR1 physically interacts with MKK4/MKK5 and negatively regulates a MAP kinase cascade to modulate plant innate immunity. *PLoS Genet.* 10:e1004389 10.1371/journal.pgen.1004389 PMC4022593 24830651

[B54] Zhao T. Rui L. Li J. Nishimura M. T. Vogel J. P. Liu N. (2015). A truncated NLR protein, TIR-NBS2, is required for activated defense responses in the exo70B1 mutant. *PLoS Genet.* 11:e1004945 10.1371/journal.pgen.1004945 PMC4305288 25617755

[B55] Zheng Z. Qamar S. A. Chen Z. Mengiste T. (2006). *Arabidopsis* WRKY33 transcription factor is required for resistance to necrotrophic fungal pathogens. *Plant J.* 48 592–605. 10.1111/j.1365-313X.2006.02901.x 17059405

[B56] Zhou N. Tootle T. L. Glazebrook J. (1999). *Arabidopsis* PAD3, a gene required for camalexin biosynthesis, encodes a putative cytochrome P450 monooxygenase. *Plant Cell* 11 2419–2428. 10.1105/tpc.11.12.2419 10590168 PMC144139

