# Supplementary figures and images for: Mutation of the Glucosinolate Biosynthesis Enzyme Cytochrome P450 83A1 Monooxygenase Increases Camalexin Accumulation and Powdery Mildew Resistance
Source: Front Plant Sci. 2016 Mar 2;7:227. doi: 10.3389/fpls.2016.00227 (PMC4774424; doi:10.3389/fpls.2016.00227)

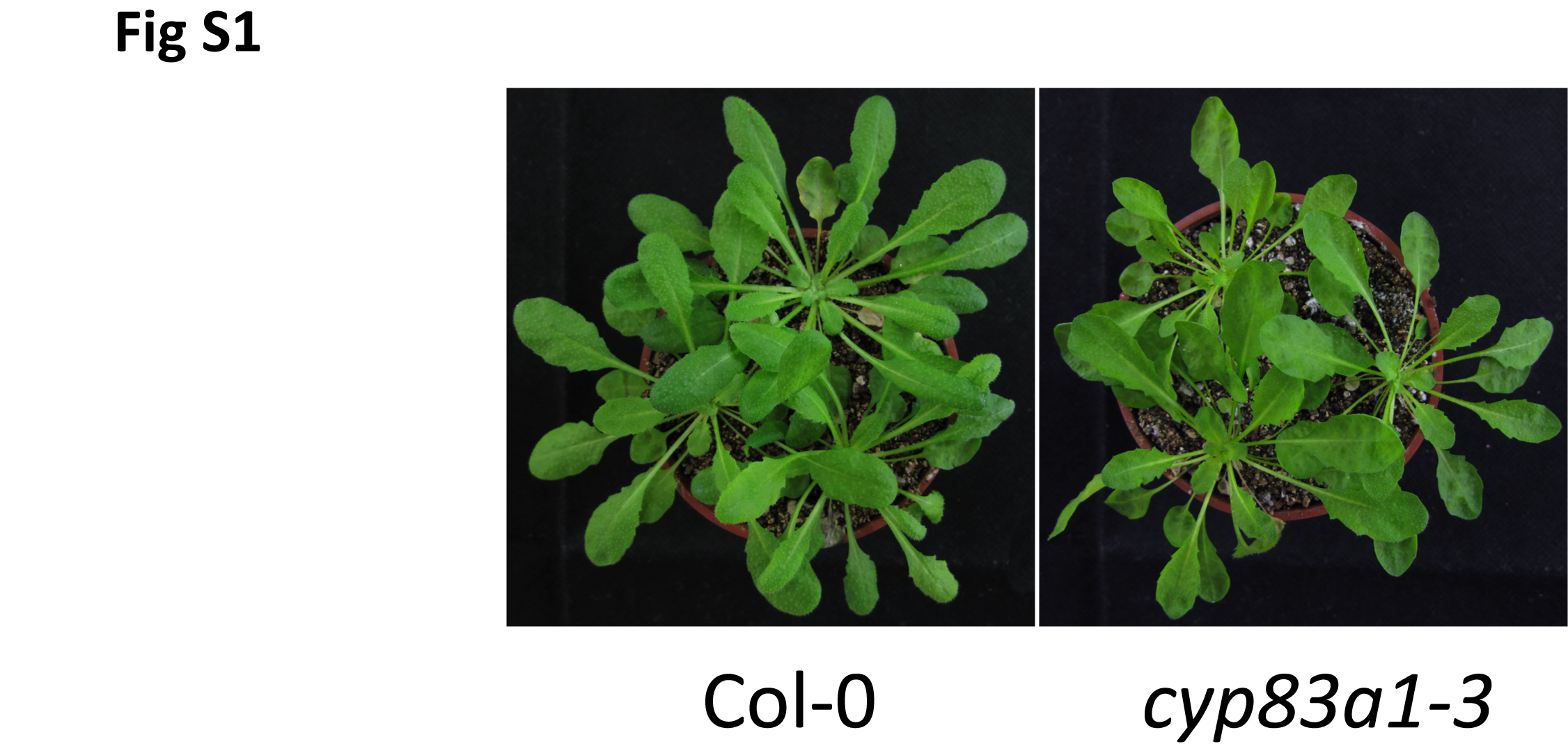

Supplement: Supplementary file 1 [file Image_1.JPEG]

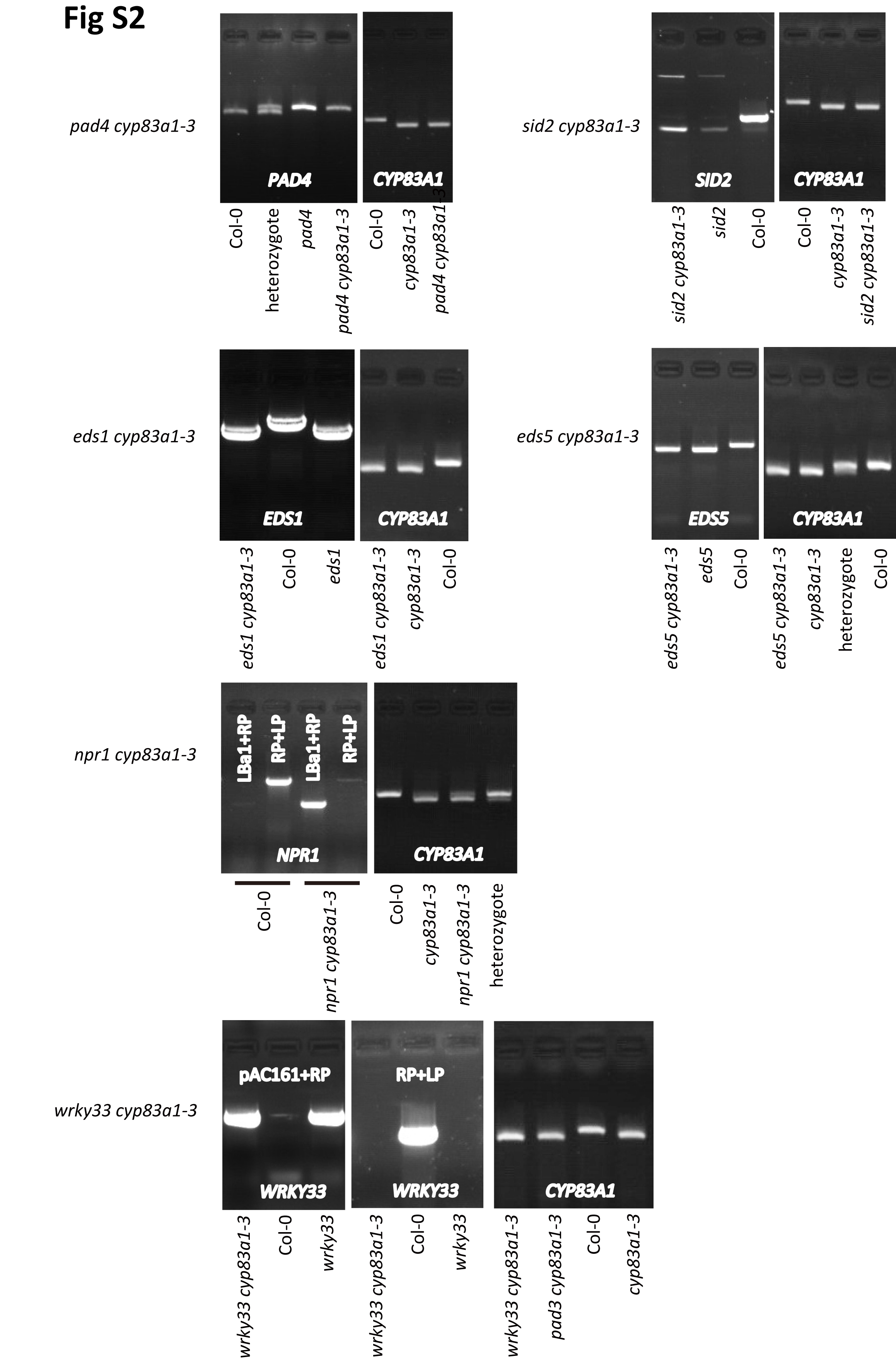

Supplement: Supplementary file 2 [file Image_2.JPEG]

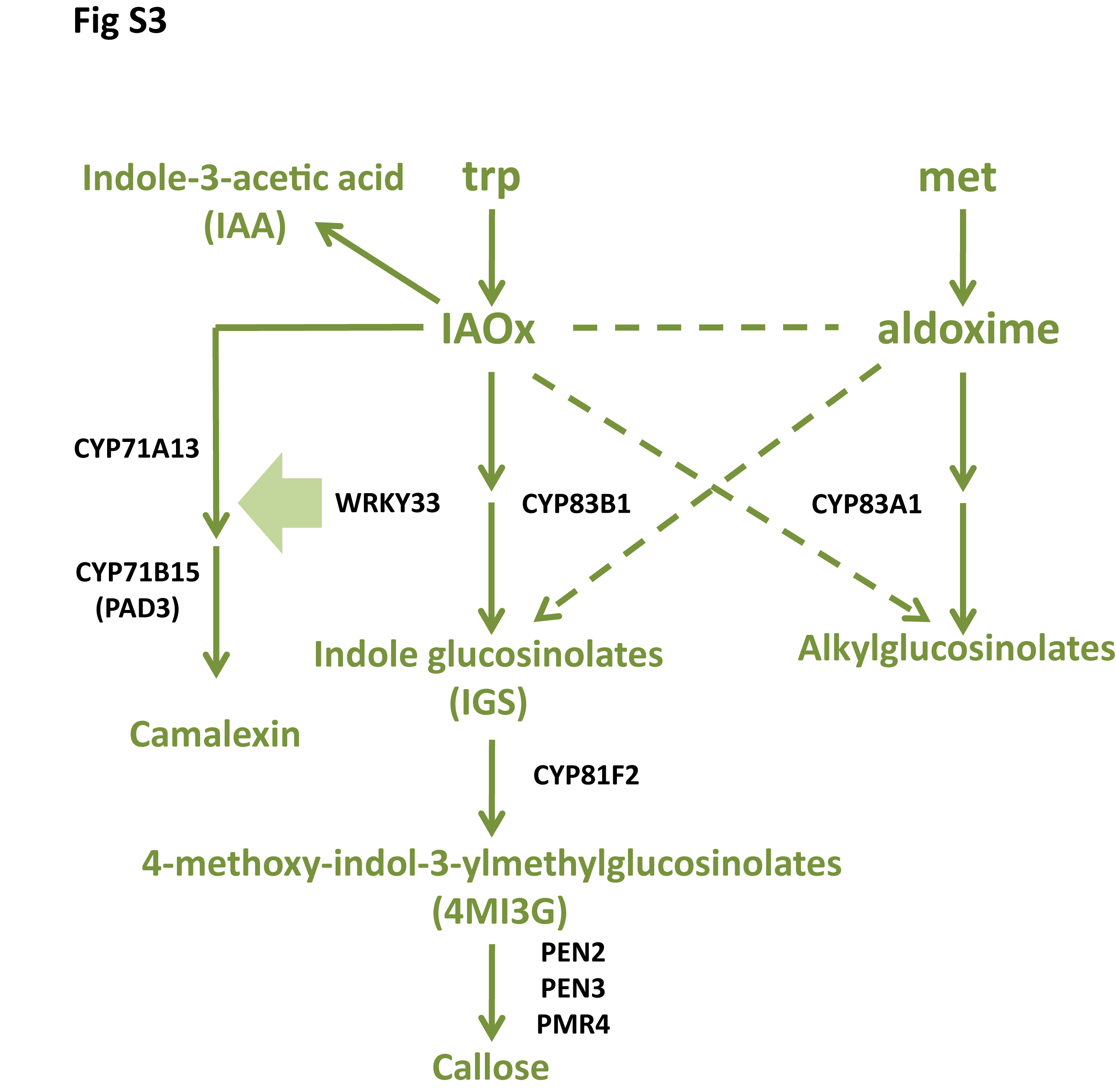

Supplement: Supplementary file 3 [file Image_3.JPEG]

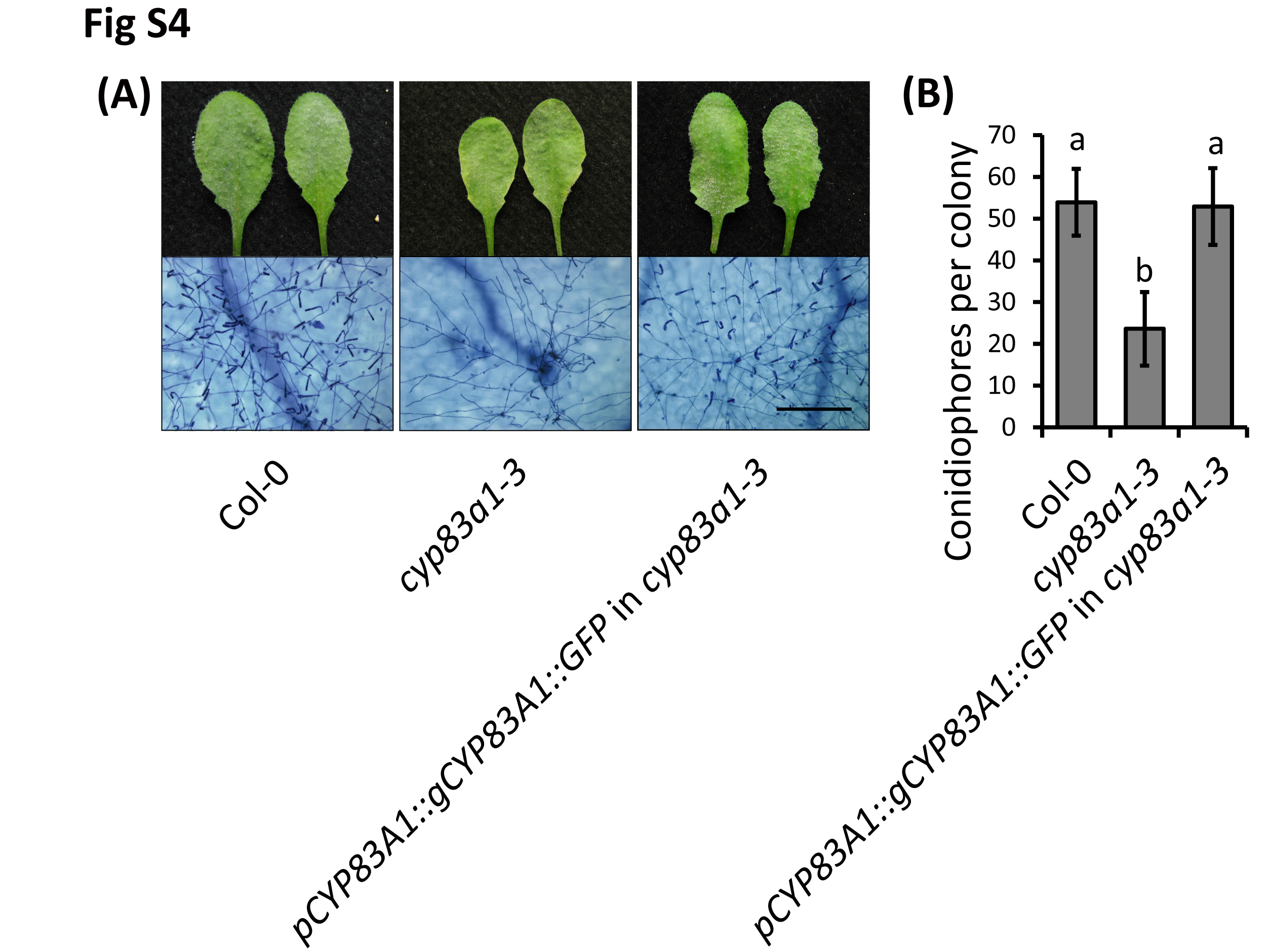

Supplement: Supplementary file 4 [file Image_4.JPEG]
