# Supplementary material for: Mutation of the Glucosinolate Biosynthesis Enzyme Cytochrome P450 83A1 Monooxygenase Increases Camalexin Accumulation and Powdery Mildew Resistance
Source: Front Plant Sci. 2016 Mar 2;7:227. doi: 10.3389/fpls.2016.00227 (PMC4774424; doi:10.3389/fpls.2016.00227)
Supplement: Supplementary file 5 [file Table_1.DOC]

**Table S1** Primers used in genotyping and gene expression analysis

| **genotyping** | | |
| --- | --- | --- |
| Primer name | Sequence (5’🡪3’) | Enzyme |
| *cyp83a1-3*-dCaps-F | ACGGACTACGAGTTTATACCGCTCG | XhoⅠ |
| *cyp83a1-3*-dCaps-R | GCTAATACTTGTTCACTTTCTCTGG |
| *wrky33-2*-LP | TCCGTTCAACGTTTGAGAGTC |  |
| *wrky33-2*-RP | GTTTGGCTCCATTGTTCTGAC |
| *pad4-1*-dCaps-F | CTGAGTTAGCCAATGAGCTTGCTAG | KpnⅠ |
| *pad4-1*-dCaps-R | TCCACCATTTTAATCACTGGGTAC |
| *sid2-2*-F | TACGAGAGAATATAAGAGAAAAGTTA |  |
| *sid2-2*-R | CAGATAGAAAGAAAAAGGGTTAAAG |
| *npr1-63*-LP | ATTTGTTTGAAGCACACCTGC |  |
| *npr1-63*-RP | CTCTCAAAGGCCGACTATGTG |
| *eds1-2-*F | AGATCAATGGCGTTTGAAGCTCTTA |  |
| *eds1-2-*R | ACCCCATCATGAGACCATTTCAATC |
| *eds5-1*-dCaps-F | ATCTGATTCTTGATATGTTTGCCG | HpaⅡ |
| *eds5-1*-dCaps-R | CGCCTGGTGAACAAGAAACAAC |
| **gene expression analysis** | | |
| *ACTIN2*-F | AGTGTCTGGATCGGTGGTTC | |
| *ACTIN2*-R | CCCCAGCTTTTTAAGCCTTT | |
| *PR1*-F | GTGGGTTAGCGAGAAGGCTA | |
| *PR1*-R | ACTTTGGCACATCCGAGTCT | |
| *PR2*-F | TCGATGAGAATAAGAAGGAACCAAC | |
| *PR2*-R | ATAACAACATACTACACGCTGAAAG | |
| *FRK1*-F | ATCTTCGCTTGGAGCTTCTC | |
| *FRK1*-R | TGCAGCGCAAGGACTAGAG | |
| *CYP83A1*-F | AGTCAAGCCCGAAACCGAGAG | |
| *CYP83A1*-R | GCAGTATCTGTTCCCGCCACTA | |
| *SUR2*-F | ACTCTTGACCCTAACCGCCCTA | |
| *SUR2*-R | TGTCAGTTCCCGGCACAACAAT | |
| *CYP71A13*-F | TGAATGGTGGACGTGATGTTGTGTT | |
| *CYP71A13*-R | TTGTTGGTGAGCAGATTGAGAATGC | |
| *PAD3*-F | ACGAGCATCTTAAGCCTGGAAG | |
| *PAD3*-R | TGCCAGCGACTCCACCAATC | |
